# Effects of Thyroid Autoimmunity on Early Atherosclerosis in Euthyroid Girls with Hashimoto’s Thyroiditis

**DOI:** 10.4274/jcrpe.2145

**Published:** 2016-06-06

**Authors:** Pınar İşgüven, Yasemin Gündüz, Mukaddes Kılıç

**Affiliations:** 1 Sakarya University Faculty of Medicine, Department of Pediatric Endocrinology, Sakarya, Turkey; 2 Sakarya University Faculty of Medicine, Department of Radiology, Sakarya, Turkey; 3 Sakarya University Faculty of Medicine, Department of Pediatrics, Sakarya, Turkey

**Keywords:** Hashimoto’s thyroiditis, carotid intima-media thickness, adolescent girls, Atherosclerosis

## Abstract

**Objective:**

In the current study, we aimed to investigate whether thyroid autoimmunity (TA) had any effect on carotid intima-media thickness (cIMT) and enhanced the risk of cardiovascular disease (CVD) independent of thyroid function (TF) in pubertal girls with Hashimoto’s thyroiditis (HT).

**Methods:**

Sixty-six newly diagnosed euthyroid girls with HT with a mean age of 14.4±2.4 years were included in the study. The control group consisted of 41 age- and body mass index (BMI)-matched healthy girls. At enrollment, all subjects underwent physical examination including blood pressure, standing height, weight, waist circumference (WC), and hip circumference measurements. The lipid profile, high-sensitivity C-reactive protein (hs-CRP), homocysteine, blood glucose, insulin, TF, and thyroid antibodies were measured, and thyroid ultrasound and cIMT were performed.

**Results:**

There were no significant differences in anthropometric variables between the two groups, but the patients with HT had significantly higher waist-to-hip ratio (WHR). Thyroid hormones, insulin, homocysteine, and homeostatic model assessment-insulin resistance were not different between the two groups. Serum hs-CRP levels were significantly higher in patients than controls (3.4 ng/mL vs. 2.03 ng/mL), (p<0.001). Patients were also characterized by significantly higher total cholesterol (166.4±27 mg/dL vs. 151±22 mg/dL), (p<0.01) and low-density cholesterol (95.8±24.4 mg/dL vs. 82.6±20.7 mg/dL), (p<0.01) levels. Patients, regardless of TF, had significantly increased cIMT compared with controls [0.28 mm vs. 0.25 mm, (p<0.001)], and cIMT was correlated with weight-standard deviation score (SDS), BMI-SDS, WC-SDS, and WHR. This increase in cIMT was associated independently with BMI-SDS and hs-CRP levels.

**Conclusion:**

TA may be related to chronic inflammation, which may cause endothelial dysfunction, a promoter of atherosclerosis in girls with HT. cIMT is a good tool for the early detection and the monitoring of early atherosclerosis in euthyroid patients with HT. Early detection of risk factors of CVD, may be helpful for planning treatment and interventions, so as to prevent complications from the disease in adulthood.

**WHAT IS ALREADY KNOWN ON THIS TOPIC?**

Hashimoto’s thyroiditis (HT) increases the intima-media thickness of the carotid artery (cIMT), regardless of thyroid dysfunction and traditional cardiovascular risk factors.

**WHAT THIS STUDY ADDS?**

Our study is the first to investigate the association between cIMT and thyroid autoimmunity in euthyroid children with HT. The importance of the current study is that although childhood is accepted as an insidious period for atherosclerosis, we found that the euthyroid girls with HT have increased cIMT.

## INTRODUCTION

As the most common organ-specific autoimmune disorder, Hashimoto’s thyroiditis (HT) is characterized by infiltration of the thyroid gland by inflammatory cells and production of autoantibodies to thyroid-specific antigens such as thyroglobulin (Tg) and thyroid peroxidase (TPO) (1). HT is associated with various degrees of thyroid dysfunction, and hypothyroidism is a well-known cardiometabolic risk factor (2). However, the influence of thyroid autoimmunity (TA) on the cardiovascular system (CVS) in the absence of overt thyroid dysfunction is still unclear.

Although atherosclerosis manifests clinically in adulthood, in recent years, it has been accepted that the disorder has a long insidious course and has its onset in childhood (3). The classical risk factors of cardiovascular disease (CVD) are accepted to be positive family history of early CVD, hypertension, obesity, hyperinsulinemia, and dyslipidemia (2).

Recently, evidence has been put forward indicating that chronic inflammation is an important pathogenic feature in atherosclerotic lesion formation. Cellular and humoral inflammatory responses are involved in the initiation and progression of atherosclerotic lesions (4). There are various inflammatory markers that have been shown to predict cardiovascular events. The high-sensitivity C-reactive protein (hs-CRP), main marker of inflammation, recently emerged as a major cardiovascular risk factor (5). High serum homocysteine concentration is also a new risk factor for atherosclerosis. The atherogenic effect of homocysteine is related to cytotoxin action on the endothelial cells and their function (6). Because inflammation causes impaired endothelium-dependent vasodilation, endothelial dysfunction (ED) could be a mechanism underlying the atherosclerosis (7). Since ED occurs early in the development of atherosclerosis, demonstration of ED could possibly lead to an early diagnosis of cardiac pathology (8).

Measurement of the carotid intima-media thickness (cIMT) of the common carotid artery is a non-invasive and effective procedure for evaluation of subclinical atherosclerosis. Increased cIMT is an indicator of early structural atherosclerosis and a strong predictor of future cardiovascular morbidity (9).

There are a number of reports in the literature that have shown the association between increased cIMT and overt or subclinical hypothyroidism in adults (10). There have also been reports regarding the impact of TA on the CVS. Ciccone et al (11) reported that autoimmunity has been associated with an increase in carotid atherosclerosis in obese women independent of thyroid function, obesity, and cardiovascular risk factors.

To the best of our knowledge, there are no data about the effects of TA on atherosclerosis among euthyroid children and adolescents with HT. In the present study, we aimed to evaluate whether TA is associated with carotid atherosclerosis and other cardiovascular risk markers in euthyroid pubertal girls with HT.

## METHODS

At the outpatient clinic of the Department of Pediatric Endocrinology of the Sakarya University Faculty of Medicine, 66 euthyroid, newly diagnosed pubertal girls with HT who were positive for TPO and/or Tg antibodies and who mostly had parenchymal heterogeneity according to thyroid ultrasound (US) were included to the study. Mean age was 14.7±2.4 (range 10-18) years. The control group consisted of 41 age- and body mass index (BMI)-matched healthy pubertal girls with negative serum thyroid autoantibodies and normal thyroid function. The girls in the control group had come to the hospital to get a report of good health. Samples were drawn between January 2015 and March 2015. For all participants, the inclusion criteria consisted of having normal serum free thyroxine (fT4) concentrations (normal range [NR]: 10.3-24.4 pmol/L) and thyroid-stimulating hormone (TSH) levels (NR: 0.3-5 µU/mL). Thyroid antibodies were considered to be positive if anti-TPO antibodies were greater than 35 IU/mL and anti-Tg antibodies were greater than 40 IU/mL, as indicated by the testing kit. Subjects with thyroid dysfunction, a history of cardiovascular or muscle disease, documented diabetes mellitus, severe dyslipidemia, any chronic or autoimmune disease other than HT, or subjects who had been on L-thyroxin treatment or any medication with a possible effect on body weight and lipid levels were excluded. All patients and controls had serum fasting glucose levels <110 mg/dL.

The study was approved by the local ethics committee, and all participants and their families provided written informed consent.

At enrollment, all subjects underwent a physical examination including blood pressure, standing height, weight, waist, and hip circumference (HC) measurements. Blood pressure was measured using an automated sphygmomanometer after the subjects had rested for nearly 10 minutes. Height was measured without shoes using a Seca 264 wireless stadiometer (UK). Weight was measured to the nearest 0.1 kg on a standard electronic scale with the subject wearing only underwear and no shoes. BMI was calculated as body weight (kg) divided by square of height (m2). With the patient in standing position, waist circumference (WC) was measured at the level of the umbilicus and HC was measured at the widest part of the gluteal region. We used population-specific data for calculation of the anthropometric values to define as standard deviation score (SDS) (12,13,14,15).

Lipid profile, hs-CRP, homocysteine, blood glucose, insulin, free triiodothyronine (fT_3_), fT_4_, TSH, anti-TPO, anti-Tg, thyroid US, and cIMT were estimated in all patients and controls.

The venous blood samples were obtained in the morning by venipuncture after overnight fasting. Plasma glucose was measured using the glucose-oxidase method. Insulin, serum fT_3_, fT_4_, TSH, anti-TPO, and anti-Tg concentrations were measured by chemiluminescent microparticle immunoassay (CMIA) method using Abbott Architect, USA kits with Abbott I2000 analyzer. Total cholesterol (TC), high-density lipoprotein-cholesterol (HDL-C), low-density lipoprotein-cholesterol (LDL-C), and triglycerides (TG) were measured with routine enzymatic methods using Abbott C 16000 analyzer. Hs-CRP (NR: 0-5 ng/mL) was measured with an immunonephelometric assay using a BNII Nephelometer (Siemens Healthcare Diagnostics, Deerfield, IL). Total plasma homocysteine (NR: 5-12 µmol/L) was measured by chemiluminescent immunoassay (IMMULITE 2000 Siemens, Healthcare Diagnostics, Germany). All assays were performed according to manufacturers’ instructions.

cIMT was determined by a real-time B-mode ultrasound (Toshiba Aplio 400, Japan) using a linear transducer (7.5-10 MHz). To avoid variations, the cIMT examination was performed by the same radiologist (Y.G.), who was blind to the subjects’ characteristics.

### Statistical Analysis

Number Cruncher Statistical System (NCSS) 2007&Power Analysis and Sample Size (PASS) 2008 Statistical Software (Utah, USA) were used for statistical analyses. Descriptive statistical methods (mean, standard deviation, median, frequency, ratio, minimum, maximum) as well as Student’s t-test for paired group comparison of normally distributed parameters and Mann-Whitney U test for paired group comparison of non-normally distributed parameters for quantitative data were used in the evaluation of the data. In the evaluation of relationships between parameters, Pearson correlation analysis was used for parameters that showed a normal distribution and Spearman’s correlation analysis for parameters that showed a non-normal distribution. The parametric data has been shown as mean ± SD and non-parametric data-as median (minimum-maximum).

Risk factors affecting cIMT were determined using the multivariate linear regression analysis. Statistical significance was set at p<0.01 and p<0.05.

## RESULTS

Anthropometric characteristics and thyroid-related values of patients with HT and controls are summarized in Table 1. There were no significant differences between the two groups in terms of anthropometric variables, except for the waist-to-hip ratio (WHR) which was significantly higher in patients with HT. There was no difference between the patients and the control group in thyroid hormone levels.

**Figure 1:**
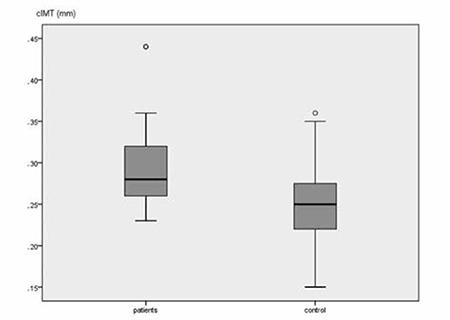
Mean carotid intima-media thickness values of patients and controls. cIMT: carotid intima-media thickness

Cardiovascular risk factors and cIMT are summarized in Table 2. Systolic and diastolic blood pressures were not different between the two groups (p>0.05). Serum fasting glucose level was significantly higher in the patient group (p<0.01). Insulin, glucose-insulin ratio, and homeostatic model assessment-insulin resistance (HOMA-IR) were not different between the groups (p>0.05). Serum hs-CRP levels were significantly higher in patients than in controls (p<0.01). Serum homocysteine levels were similar between the two groups (p>0.05). Patients were also characterized by significantly higher TC and LDL-C values than the controls (p<0.01). As seen in Figure 1, cIMT was increased significantly in the patient group as compared to the control group (p<0.01). None of the patients and controls had atherosclerotic plaques.

cIMT was positively correlated with body weight SDS (r=0.39; p<0.001), WC SDS (r=0.41; p<0.001), BMI SDS (r=045; p<0.001), WHR (r=0.37; p<0.003), and hs-CRP (r=0.39; p<0.003) in the patient group. There were no correlations between cIMT and thyroid-related markers in either group.

The multiple regression analysis has shown that only hs-CRP and BMI SDS were the independent variables for cIMT in girls with HT (Table 3).

## DISCUSSION

It is well established that hypothyroidism is associated with a higher risk for CVD. cIMT measurement is an effective and non-invasive procedure for evaluation of cardiovascular risk. Increased cIMT has been reported in patients with overt or subclinical hypothyroidism compared to healthy controls (16). HT is the most common cause of hypothyroidism, but it is not clear if TA is a risk factor for atherosclerosis independent of thyroid function. The pathogenic mechanisms underlying the increased cIMT in euthyroid patients with HT have not been studied extensively. This study has shown that euthyroid pubertal girls with HT, regardless of thyroid function, have significantly increased cIMT compared to healthy controls.

It is well known that hypercholesterolemia is one of the major risk factors of atherosclerosis and thus of increased cIMT (17). There is a close association between thyroid function and dyslipidemia in overt hypothyroidism (18). Autopsy findings in children and adolescents with hypothyroidism have demonstrated an increase in atherosclerotic lesions in the coronary artery with increasing TC and LDL-C and decreasing HDL-C (19). Because of diminished number of LDL-C receptors in the liver, the fractional excretion of LDL-C is reduced in hypothyroidism (20). In our study, even though fasting TC and LDL-C levels were within normal limits, they were higher in the patients than in the controls. Although elevated TSH is suggested as a major factor in the dyslipidemia (21), in the present study, there was no difference in mean TSH levels between patients and controls. The reason for the differences between lipid values was not clear.

Increased cIMT has been reported in children with diabetes, hypertension, and childhood obesity (22). The common pathogenetic factor involved in endothelial damage in obese and diabetic children and adolescents seems to be reduced insulin function. Insulin acts by modulating the release of vasodilator substances (i.e. nitric oxide and prostaglandins) from the vascular endothelium (10). In the current study, fasting serum glucose levels were elevated in the patients compared to the controls, but serum fasting insulin, glucose-insulin ratio, and HOMA-IR were not different between the groups. Also, no relationship was found between cIMT and HOMA-IR.

The harmful effects of elevated glycemia and mild dyslipidemia on the vasculature, affecting endothelial function are well documented (22). However, insulin function and fasting TC and LDL-C levels were within normal limits in our patients. Therefore, these harmful effects on the vasculature might not be significant.

On the other hand, individuals who suffer from systemic autoimmune diseases like rheumatoid arthritis and systemic lupus erythematosus also have a greater and earlier incidence of atherosclerotic CVD (23). Therefore, as an autoimmune disease, HT itself may be responsible for autoimmune, inflammation-based ED. Taddei et al (24) demonstrated in adult patients with HT that ED comes only from the autoimmune disease, independent from other cardiovascular risk factors. In another study from Turkey, it was demonstrated that TA may have some effect on hyperlipidemia, obesity, and abdominal obesity independent of thyroid function (25). Topaloglu et al (26) have found that euthyroid, premenopausal women with HT have increased cIMT independent of the thyroid function tests. In the Rotterdam study, a greater incidence of atherosclerosis was observed in anti-TPO-positive hypothyroid patients (27). These observations suggest an atherogenic role of thyroid antibodies. A mechanism explaining this link may be a state of chronic inflammation in thyroid antibody-positive patients, which causes ED, ultimately resulting in atherosclerosis (28).

According to Volpe’s (29) hypothesis, in HT, helper T cells are not suppressed because of defective suppressor T cells, and thus are able to produce a lot of cytokines such as interferon gamma, interleukin-2, and tumor necrosis factor alpha. Sultan et al (30) suggested that these cytokines might cause weight gain and hyperlipidemia. Therefore, it is likely that the higher serum levels in glucose and lipids are at least in part explained by TA in these patients.

Factors involved in inflammatory processes may be important determinants of increased cIMT, including hs-CRP and homocysteine (26). The predictive association between CRP and coronary artery disease has been extensively confirmed (31). Kaptoge et al (32) in a meta-analysis of 160,309 patients without antecedents of CVD found that hs-CRP concentration shows a continuous association with the risk of coronary disease and cardiovascular mortality. In the present study also, hs-CRP levels were higher in patients than in controls and there was a positive correlation between hs-CRP and cIMT in the patient group. CRP may contribute to inflammation in atheroma and also may be actively involved in early atherogenesis. It has been shown that native CRP deposition displays calcium-dependent in vitro binding to LDL in the arterial wall and induces classical pathway of complement activation, and that stimulation of monocyte chemotaxis and inhibition of neutrophil chemotaxis may be important inflammatory mechanisms (31).

Several studies have demonstrated hyperhomocystinemia in overt hypothyroidism, with serum fT4 being an independent determinant of homocysteine concentrations (6). In the present study, there was no significant difference between the homocysteine levels of the two groups. This might be due to the fact that both groups consisted of euthyroid girls.

Kollias et al (33) examined the association between cIMT and several cardiovascular risk factors in 448 apparently healthy adolescents, and central obesity and systolic blood pressure appeared to be independently associated with cIMT. While BMI remains the most commonly used obesity measure, its main limitation compared to WC and WHR is that it does not take into account body fat distribution (34). Findings of previous studies comparing the ability of BMI and measures of abdominal obesity to identify cardiometabolic risk factors are conflicting. WC has been found to be related to cardiometabolic risk factors independent of BMI in some studies (35), while other studies have found that WC and WHR are not better than BMI for the identification of cardiometabolic risk (36). Previous studies done among Swedish and Turkish children showed that BMI was a better predictor of WHR and of skinfolds (37,38). In our study, the patients had higher WHR, which points to central obesity. The cIMT was significantly correlated with WC-SDS, BMI-SDS, and WHR, but among them, only BMI-SDS was the independent variable for the cIMT in girls with HT.

Our study is the first to investigate the association between cIMT and TA in euthyroid children with HT. The importance of the current study is that although childhood is accepted as an insidious period for atherosclerosis, we found that the girls with HT have increased cIMT.

In conclusion, in girls with HT, TA may be responsible for chronic inflammation, which may cause ED, a promoter of atherosclerosis. cIMT is a good tool for the early detection and the monitoring of early atherosclerosis in euthyroid patients with HT. Hs-CRP level and BMI can be early leading indicators for cardiovascular risk in young girls with HT. The combination of these factors with biochemical markers like hypercholesterolemia and hyperglycemia may play an important role in the prediction of cardiovascular risk in young girls with euthyroid HT. Early detection of risk factors of CVD may be helpful for planning treatment and interventions, so as to prevent complications from the disease in adulthood. Finally, further longitudinal studies are needed to help establish the link between accelerated atherosclerosis and autoimmunity during childhood.

## Ethics

Ethics Committee Approval: The study was approved by the local ethics committee December 2014, Informed Consent: Informed consent was obtained.

Peer-review: External peer-reviewed.

## Figures and Tables

**Table 1:**
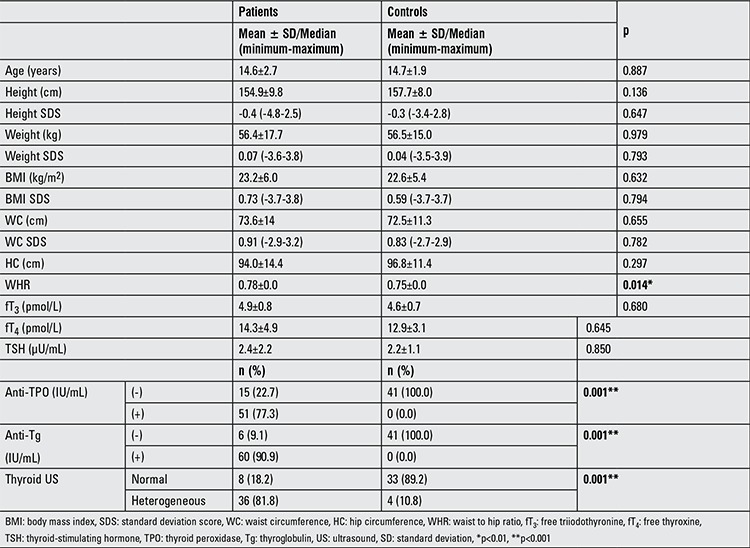
Anthropometric characteristics, thyroid antibody levels, and results of thyroid function tests and thyroid ultrasonography in the patients and in controls

**Table 2:**
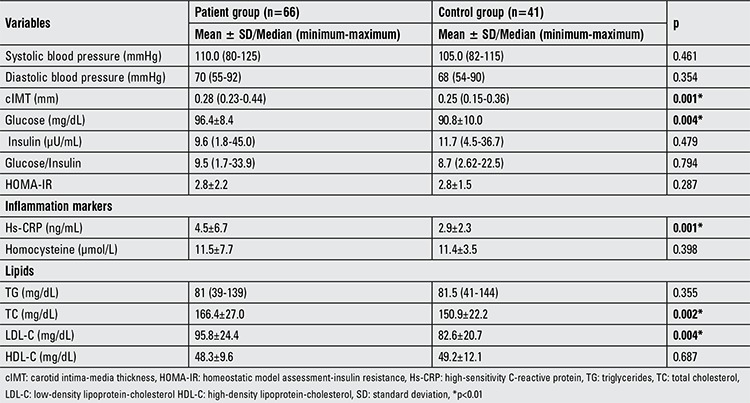
Differences in the variables analyzed in the patient and control groups

**Table 3:**
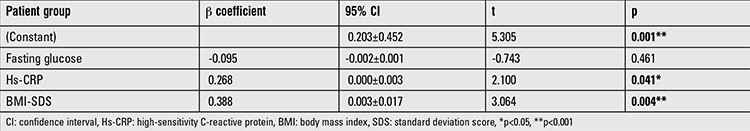
The multiple regression analysis coefficients between carotid intima-media thickness and the other parameters in the patient group
